# Advances in ORR Catalysis Promoted by Graphene-Supported Low-Cost Metal Clusters: A DFT Study

**DOI:** 10.1021/acsami.5c08441

**Published:** 2025-06-16

**Authors:** Ida Ritacco, Giuseppe Santoriello, Gianluca Gatta, Matteo Farnesi Camellone, Lucia Caporaso

**Affiliations:** † Dipartimento di Chimica e Biologia, 19028 Università degli Studi di Salerno, via Giovanni Paolo II 132, Fisciano, Salerno 84084, Italy; ‡ 19040 CNR-IOM, Consiglio Nazionale delle Ricerche - Istituto Officina dei Materiali, c/o SISSA, Trieste 34136, Italy; § Dipartimento di Medicina di Precisione Divisione di Radiologia, Università della Campania Luigi Vanvitelli, Napoli 80131, Italy

**Keywords:** oxygen reduction reaction (ORR), heterogeneous catalysis, clusters, graphene, DFT, N-doped graphene, theoretical overpotential (η)

## Abstract

The oxygen reduction reaction (ORR), which converts molecular oxygen (O_2_) into water (H_2_O), is critical for renewable energy transformation processes. However, its industrial application is hindered by long conversion times. Recent studies suggest that transition metal clusters deposited on graphene are promising candidates for ORR catalysis. In this work, we employed density functional theory (DFT) calculations to explore the thermodynamically most stable energy profile of the ORR on pentamer metal clusters (Fe_5_, Co_5_, and Pt_5_) supported on undoped graphene and nitrogen-doped graphene (for Fe_5_), under standard electrochemical conditions (pH = 0 and *U* = 0). Both the “standard” intermediates (*OOH, *O, *OH) and the “unconventional” intermediates (*O*OH, *OH*OH) were studied, analyzing thermodynamic stability, adsorption energies, and the influence of the implicit water solvent. Our results reveal that the inclusion of “unconventional” intermediates significantly alters the reaction thermodynamics, presenting a new pathway that is energetically more favorable than the classical one. Catalytic performance predictions, based on the theoretical overpotential (η^ORR^), indicate that the four catalysts exhibit good stability and high activity in both reduction mechanisms. In particular, Fe_5_@NGr shows the best catalytic performance in the “unconventional” mechanism, with an η^ORR^ close to zero. This study, for the first time, demonstrates how the metal cluster and the support’s electronic and structural properties influence the stability of ORR intermediates and catalytic performance. The improved performance of Fe_5_@NGr in the “unconventional” mechanism highlights the importance of selecting the right metal and engineering the graphene support, particularly through N-doping, for the rational design of low-cost, high-performance catalysts.

## Graphical abstract

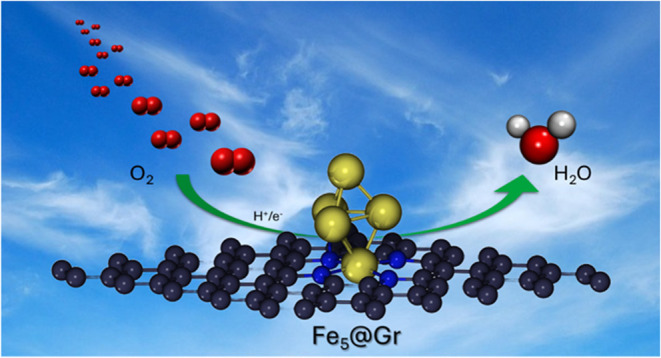

## Introduction

In electrochemistry, the oxygen reduction reaction (ORR) is a crucial and intricate process that plays a key role in different energy conversion technologies, from fuel cells to metal-air batteries.^1,2^

Since the research moves toward sustainable and clean energy sources and since the oxygen present in our atmosphere has immense potential as an electron acceptor in electrochemical devices, thus contributing to the electricity production with minimal environmental impact, understanding the complexity of the ORR becomes fundamental.

The oxygen reduction reaction is a key electrochemical process that converts molecular oxygen (O_2_) into water (H_2_O) via a four-electron mechanism, typically proceeding through a series of complex and interconnected steps involving the formation of the “standard” *OOH, *O, and *OH intermediates, commonly referred to as the “classical” mechanism. Despite its importance, the oxygen reduction process has limitations, such as high overpotentials and slow kinetics,^3^ which hinder the development of efficient and economically sustainable energy conversion technologies.^4^ Therefore, an in-depth understanding of the mechanisms governing ORR is indispensable for the design, modeling, and synthesis of target electrocatalysts.

In this contest, graphene-supported materials are promising ORR catalysts as they are inexpensive and exhibit peculiar structural and electronic characteristics.^5^ According to previous experimental and theoretical studies,^6–12^ graphene is an ideal candidate for the ORR catalysis due to its two-dimensional hexagonal honeycomb structure, which provides it with a large surface area, and its high thermal and electronic conductivity.^13^ These characteristics make it an excellent catalytic support. In fact, the adsorption and/or integration of transition metal clusters in the graphene honeycomb structure introduces catalytically active sites^14^ that could significantly improve the ORR kinetics. The combination of the unique properties of graphene together with those of transition metal clusters generates an advantageous synergistic effect that allows to overcome the limitations associated with conventional ORR catalysts. To date, graphene-supported platinum clusters (Pt_n_) are among the most widely used catalysts for the molecular oxygen reduction.^15^ However, since platinum is a noble metal, the costs related to the Pt_n_-graphene catalyst production are high. In addition, the catalytic activity of the Pt clusters supported on a graphene monolayer is strongly affected by the applied potentials, suggesting a high sensitivity of these systems to experimental conditions.^16^ Therefore, to break through the limits related to the Pt_n_-graphene catalysts, it is necessary to focus the research on the study of low-cost transition metal clusters that are highly active in the molecular oxygen reduction process.

The aim of this work was to investigate through DFT calculations the most thermodynamically stable ORR processes on pentamer metal clusters (*M*_5_) in order to correlate the chemical characteristics of the metals with their catalytic performances.

Inspired by the insights provided by Di Liberto et al. on single-atom catalysts,^17^ we performed a systematic study on graphene-supported metal clusters, investigating not only the “standard” ORR intermediates but also potential “unconventional” species which could significantly impact the reaction thermodynamics.

Specifically, we computed the energy profiles of Fe_5_, Co_5_, and Pt_5_ clusters supported on undoped graphene and on nitrogen-doped graphene in the case of Fe_5_, interacting with a single O_2_ molecule under standard electrochemical conditions (pH = 0, *U* = 0). To more accurately describe the adsorption energies of both “standard” and “unconventional” intermediates, we included the effect of the implicit water solvent. Finally, catalytic performance predictions for each system, denoted for clarity as *M*_5_@Gr­(NGr), were carried out both in the gas phase and implicit solvent, based on a quantitative evaluation of the theoretical overpotential (η^ORR^).

## Computational Details

DFT calculations were performed using Quantum Expresso code^18^ employing the Perdew–Burke–Ernzerhof (PBE) exchange-correlation functional based on the generalized gradient approximation (GGA)^19^ and ultrasoft pseudopotentials.^20^ The spin-polarized Kohn–Sham equations were solved in the plane-wave pseudopotential framework, with the wave function basis set and the Fourier representation of the charge density being limited by kinetic cutoffs of 50 and 500 Ry, respectively.

The calculations were carried out using a 6 × 6 graphene (Gr) supercell, with 72 carbon atoms, and pentamer clusters of Fe, Pt, and Co (i.e., *M*_5_ = Fe_5_, Pt_5_, and Co_5_). The size and the typical motifs of the clusters used in this work represent the most stable configurations on graphene among all those possible, according to the literature.^16,21–23^

Since metal clusters (*M*_n_) must interact with both the support and the adsorbates, we chose to model clusters with more than four atoms in order to ensure that, after adsorption on graphene, metal sites would be available for interaction with the various adsorbates. In this regard, previous theoretical studies on the energy stability of Fe_n_ and Co_n_ clusters (*n* = 2···7) adsorbed on graphene^22,23^ have shown that the adsorption energy of Fe_5_ and Co_5_ on graphene is higher than that of the corresponding metal clusters with *n* = 6 and *n* = 7. This suggests: (i) a stronger interaction between these clusters and the support and (ii) a higher stability of the resulting catalysts. Furthermore, the nucleation of Fe_5_ and Co_5_ is more stable than that of larger clusters, indicating a lower propensity for Fe_5_ and Co_5_ to undergo uncontrolled growth and aggregation.^22,23^ Additionally, Fe_5_ adsorption causes less pronounced structural distortions in the graphene monolayer compared to Fe_6_ and Fe_7_.^22^ Based on these observations, we modeled pentamer clusters of Fe and Co, using the same size for the Pt cluster (Pt_5_) in order to compare the ORR results.

In the presence of N-doped graphene (NGr) as a support, the electrocatalytic performance of Fe atom in the oxygen reduction reaction (ORR) is superior to that of Co and other transition metals, such as Cu and Ni.^24,25^ This prompted us to investigate the adsorption of the Fe_5_ cluster on the NGr monolayer to better understand the influence of the nitrogen dopant on the ORR process.

Between consecutive monolayers, a vacuum separation of 18 Å along the *z* direction was entered to prevent mutual influence with the periodic images, and during the optimizations, all atoms in the supercell are allowed to fully relax. A k-point sampling of 6 × 6 × 1 Monkhorst–Pack grids in the first Brillouin zone of the supercell and a Gaussian smearing with a width of σ = 0.05 eV are used in the calculations. The van der Waals (vdW) interactions were explicitly considered employing the zero damping DFT-D3 method of Grimme.^26^ In addition, the dipole-correction method, which introduces a correcting dipole in the vacuum region, was applied to decouple the simulation cell electrostatically from its periodic images.^27,28^ The effects of the implicit water solvent on the oxygen reduction reaction catalyzed by the four different supports were evaluated performing DFT-based SCF calculations by using the ENVIRON module^29^ of Quantum ESPRESSO.^18^ For the implicit solvent, we used the SCCS formulation of the dielectric cavity^29–31^ as implemented in ENVIRON with the chosen meta-parameters tuned for correct solvation energetics of explicit H_2_O in the implicit model (env_static_permittivity = 80, env_pressure = −0.36 GPa, env_surface_tension = 47.9 dyn/cm).

The binding energy (*E*_b_) of the pentamer metal clusters *M*_5_ on Gr­(NGr)—*M*_5_@Gr­(NGr)—was computed using the equation

1 $$E_{b}=E_{M5@\mathrm{Gr}\left(\mathrm{NGr}\right)}-E_{\mathrm{Gr}\left(\mathrm{NGr}\right)}-E_{M5}$$

where *E*_*M*5@Gr(NGr)_ is the total energy of the cluster supported on the graphene­(N-doped graphene) monolayer, and *E*_Gr(NGr)_ and *E*_*M*5_ refer to the energies of the support and the isolated metal cluster, respectively. In order to gain more information about the stability of the *M*_5_@Gr­(NGr) catalysts, the cohesive energy (*E*_c_) for the pentamer metal clusters on the undoped and N-doped graphene was defined with the equation

2 $$E_{c-\mathrm{bound}}=\left(E_{M5@\mathrm{Gr}\left(\mathrm{NGr}\right)}-E_{\mathrm{Gr}\left(\mathrm{NGr}\right)}-nE_{M}\right)/n$$

where *E_M_* is the energy of a single *M* atom and *n* is the number of the *M* atom in the cluster. Therefore, *E*_c‑bound_ measures the energy stability of the *M*_5_ formation on Gr­(NGr) starting from the single metal gas phase. For comparison, the cohesive energy of the pentamer metal clusters in the free form, computed with the equation

3 $$E_{c-\mathrm{free}}=\left(E_{M5}-nE_{M}\right)/n$$

was also calculated. Generally, a more negative value of *E*_c_ indicates a more stable structure.

The charge transfers are very important to understand the interaction between the substrates, the metal clusters, and the support. Therefore, the charge analysis was performed following Bader’s theory since the charge enclosed within the Bader volume can be considered a good approximation of the total electronic charge of an atom.^32–34^ The differences between the Bader charges of the coordinated and gas phase pentamer metal clusters (Δ*q*, e^–^) were calculated according to the equation

4 $$\Delta q=\sum q\left(M_{5}@\mathrm{Gr}\left(\mathrm{NGr}\right)\right)-\sum q\left(M_{5}\right)$$

where Σ*q*(*M*_5_@Gr­(NGr)) is the sum of the Bader charges of *M*_5_ adsorbed on Gr­(NGr) monolayer and Σ*q*(*M*_5_) is the sum of the free metal clusters.

The free energy diagrams of the ORR intermediates along the electrochemical reaction pathway (see Section 1 of the Supporting Information (SI)) were calculated with the computational hydrogen electrode (CHE) model developed by Nørskov et al.^35^ according to which in standard conditions of pH = 0 and *U* = 0, where *U* is the applied electrochemical potential, the free energy of (H^+^ + e^–^) is set to half of the chemical potential of the gas phase H_2_. The difference between the free energies of the initial and final states is computed with the equation

5 ΔG=ΔE+ΔZPE−TΔS

where Δ*E* corresponds to the reaction energy obtained through DFT calculations, ZPE is the zero-point energy, *S* is the entropy, and *T* is the room temperature (298.15 K). The ZPE corrections were obtained from the vibrational frequencies calculated by treating the 3N degrees of the substrates. In the calculation of the ZPE corrections, whose values are reported in Table S1, only the substrates vibration modes are considered, whereas the catalyst is considered fixed. Based on this approximation, the vibrational contribution of the catalysts to the free energy is negligible.^36,37^ Similarly, the entropic contribution (S) of each intermediate can be neglected when compared to the same of gases.^35,38^ Therefore, only the entropy terms of the gas phase species (H_2_, O_2_, and H_2_O) were considered and taken from online international tables (see Table S2).

## Results and Discussion

### Stability and Structure of the Pentamer Metal Clusters *M*_5_ on Graphene and N-Doped Graphene

Before studying the ORR reactivity on *M*_5_@Gr­(NGr) catalysts, the stability of the pentamer clusters of Fe, Pt, and Co on the undoped and, for Fe_5_, N-doped graphene monolayers was investigated.

As described in the Computational Details section, the initial adsorption configuration of Fe_5_, Pt_5_, and Co_5_ was chosen based on the literature data. Accordingly, the most stable structure of Fe_5_ is a trigonal bipyramid with (i) two edge metals in contact with Gr and (ii) one point embedded in the four nitrogen atoms of the NGr monolayer (Figure 1a,b, respectively).^21,22^ For Co_5_, the most stable structure consists of a trigonal pyramid with three metals of one face in contact with Gr (Figure 1c),^23^ while for Pt_5_ the most stable one is the planar side-capped square structure with two of the four atoms of the square in contact with the carbon atoms of Gr (Figure 1d).^16^

**Figure 1:**
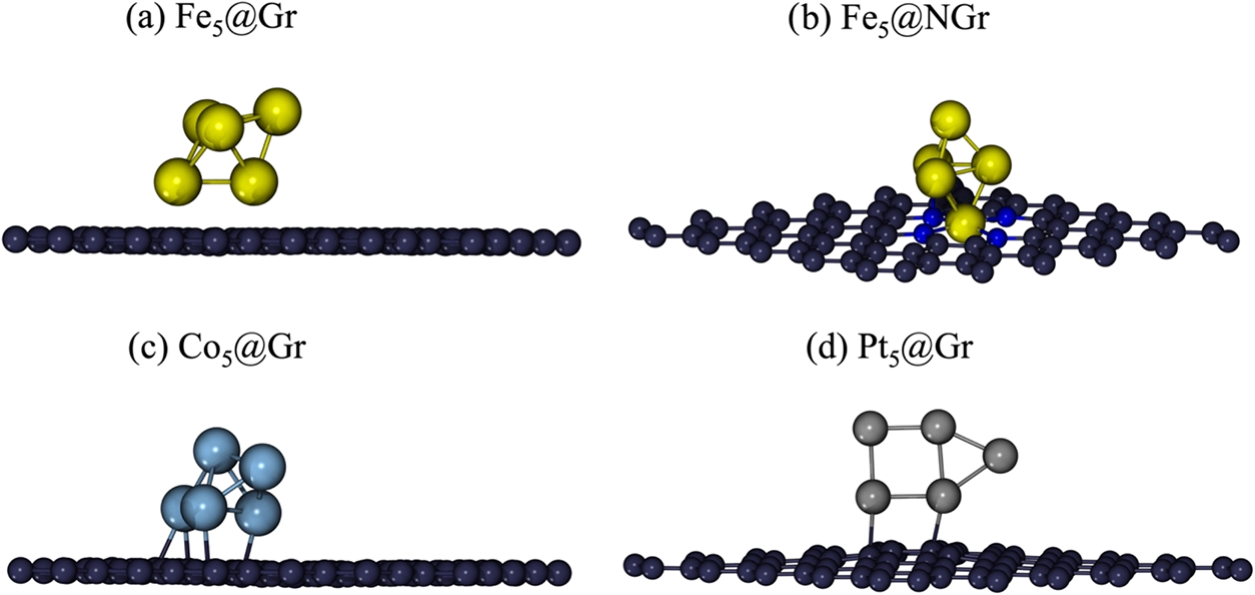
Optimized structure of (a) Fe_5_@Gr, (b) Fe_5_@NGr, (c) Co_5_@Gr, and (d) Pt_5_@Gr. Fe, Co, Pt, N, and C atoms are represented in ball and sticks and depicted in yellow, cyan, silver, blue, and gray, respectively.

To gain more insights into the interaction between *M*_5_ clusters and the undoped and N-doped graphene, the binding (*E*_b_) and cohesion (*E*_c_) energies of the clusters in free (*E*_c‑free_) and bound (*E*_c‑bound_) form were calculated according to eqs 1, 2, and 3 (see the Computational Details section) and reported in Table 1.

**Table 1:** *E*_b_, *E*_c‑free_, and *E*_c‑bound_ in eV for *M*_5_ Clusters in Free and Gr­(NGr)-Bound Form^a^

	*E*_b_	*E*_c‑free_	*E*_c‑bound_
Fe_5_@Gr	–1.4 (−1.7)	–2.0 (−2.1)	–2.3 (−2.5)
Fe_5_@NGr	–6.5 (−6.4)	–2.0 (−2.2)	–3.3 (−3.4)
Co_5_@Gr	–1.3 (−1.5)	–2.2 (−2.3)	–2.5 (−2.6)
Pt_5_@Gr	–2.1 (−2.2)	–2.6 (−2.7)	–3.0 (−3.1)

a The values are reported in eV, the energies in solvent are in parentheses.

The values of the binding energy (*E*_b_) and the cohesion energy in the bound form (*E*_c‑bound_), larger than the free ones (*E*_c‑free_), indicate the formation of stable *M*_5_@Gr­(NGr) catalysts and a stable growth of the pentamer metal clusters on the Gr­(NGr) support both in the gas phase and in the water environment (see Table 1). Although *E*_b_ and *E*_c‑bound_ are thermodynamically favored for all clusters (*E*_b_ and *E*_c‑bound_ < 0), the best interactions occur in the Fe_5_@NGr catalyst, with an *E*_b_ of −6.5 (−6.4) eV and an *E*_c‑bound_ of −3.3 (−3.4) eV (Table 1), in which one of the five Fe atoms of the cluster is directly embedded in the N-doped graphene monolayer, coordinating four nitrogen atoms (Figure 1b).

The charge difference between the three transition metals considered in this work (Fe, Co, and Pt) could play an important role in the stabilization of the systems and in the ORR. Therefore, the Bader charges (Δ*q*, e^–^) of the *M*_5_ clusters adsorbed on Gr­(NGr) were computed according to eq 4 (see the Computational Details section) and compared with those obtained for the free-form pentamer metal clusters (see Table 2).

**Table 2:** Sum of the Bader Charges (Σ*q*, e^–^) of the *M*_5_ Clusters in the Free and Gr­(NGr)-Bound Form^a^

	Fe_5_ free	Fe_5_@Gr	Fe_5_@NGr	Co_5_ free	Co_5_@Gr	Pt_5_ free	Pt_5_@Gr
Σ*q*	80.0	79.2	78.2	85.0	84.2	50.0	49.98
Δ*q*		–0.8	–1.8		–0.8		–0.02

a Δ*q* represents the difference between the Bader charges of the coordinated and gas phase clusters.

Table 2 shows that following adsorption, there is a charge transfer from the metal clusters to the support of 0.8 |e^–^| for Fe_5_ and Co_5_ and of only 0.02 |e^–^| for Pt_5_ adsorbed on Gr. Excitingly, in the case of NGr, the charge transfer from Fe_5_ increases up to 1.8 |e^–^|, indicating a further stabilization of the catalyst in terms of *E*_b_ and *E*_c_ when the metal cluster is embedded within the support, interacting via its suitable orbitals with nitrogen dopants.

This significant charge transfer leads to the electronic depletion of the metal pentamer clusters, particularly Fe_5_ and Co_5_. Once adsorbed onto the graphene support—which acts as a true “electronic sponge”—these clusters acquire a more positive charge than Pt_5_. This effect clearly highlights the key role of the graphene monolayer, which serves not only as a structural support but also as an active modulator of the electron density in the adsorbed metal clusters, thereby enhancing their efficiency as catalytic sites for the ORR. Furthermore, the greater orbital availability of Fe (3d^6^) and Co (3d^7^) atoms compared to Pt (5d^9^) provides Fe_5_ and Co_5_ clusters with a stronger ability to stably interact with O_2_ molecules. This facilitates oxygen adsorption and activation, making Fe_5_ and Co_5_ more active and efficient catalytic sites than Pt_5_ in the ORR reduction process.

### Thermodynamic Stability of the ORR Intermediates on *M*_5_@Gr­(NGr) Catalysts and Energy Reaction Paths

The intermediate species generated during the ORR play a key role in the oxygen reduction mechanism. Therefore, the first step of this work was to investigate the adsorption of the “standard” reaction intermediates for the four-electron ORR mechanism, *OOH, *O, and *OH, and the “unconventional” ORR intermediates,^17^ *O*OH and *OH*OH, on *M*_5_@Gr­(NGr) catalysts (*M*_5_ = Fe_5_, Co_5_ and Pt_5_) in relation to the electronic and structural properties of the different clusters considered.

The reduction of molecular oxygen to water proceeds through the 4-electron reaction *O*_2_(*g*) + 4H^+^ + 4e^–^ ⇋ 2H_2_O­(l), describing an associative mechanism. According to eqs 6, 7, 8, and 9, the first ORR intermediate involved in a “classical” mechanism consists in the adsorption of an OOH group on the catalytic site, which then converts to O and subsequently to OH, releasing H_2_O:

6 *+O2+H++e−→O*OH

7 O*OH+H++e−→O*+H2O

8 O*+H++e−→O*H

9 O*H+H++e−→*+H2O

According to the previously reported theoretical studies,^17^ the formation of the “standard” intermediates *OOH and *O can compete with that of the “unconventional” intermediates *O*OH and *OH*OH, respectively. Therefore, eqs 6, 7, and 8 of the classical ORR are replaced by eqs 10, 11, and 12:

10 *+O2+H++e−→O*O*H

11 O*O*H+H++e−→O*HO*H+H2O

12 O*HO*H+H++e−→O*H

Regardless of the intermediates type, the last reduction reaction step proceeds through the “standard” intermediate *OH (eq 9).

In all equations, the symbol * represents the catalytic site (*M*_5_@Gr­(NGr)), while the symbols *OOH, *O*OH, *OH*OH, *O, and *OH indicate the substrates chemisorbed on the catalysts.

Figure 2 shows the most stable optimized geometries related to the adsorption of one O_2_ molecule on the catalysts Fe_5_@Gr, Fe_5_@NGr, Co_5_@Gr, and Pt_5_@Gr with the corresponding Gibbs energy variations (Δ*G*, eV) (panels a-d, respectively, Tables 3 and 4) computed by using the equations reported in Section 1 of the SI.

**Figure 2:**
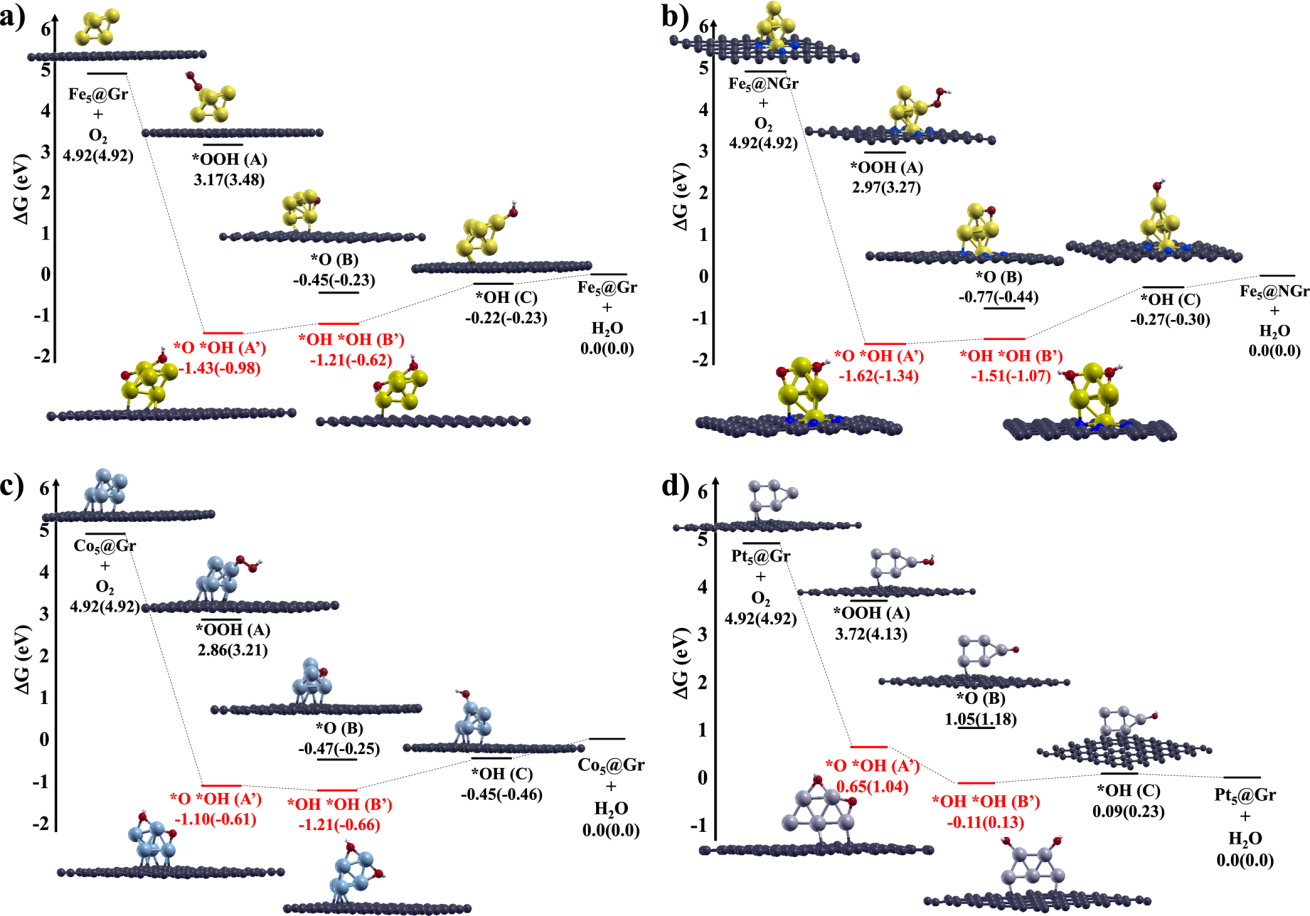
Steps and optimized structure of the “standard” (*OOH (A), *O (B), and OH (C), black lines) and “unconventional” (*O*OH (A′) and *OH*OH (B′), red lines) intermediates involved in the reduction of O_2_ by using as catalyst (a) Fe_5_@Gr, (b) Fe_5_@NGr, (c) Co_5_@Gr, and (d) Pt_5_@Gr. The Gibbs energy variations (Δ*G*) in gas phase, and in brackets in water implicit solvent, are reported in eV. Fe, Co, Pt, N, O, H, and C atoms are represented in ball and sticks and depicted in yellow, cyan, silver, blue, red, white, and gray, respectively.

**Table 3:** Bond Distance, in Å, between the Active Sites of the Pentamer Metal Clusters and the Adsorbates in the “Standard” Intermediates *OOH, *O, and *OH^a^

**1 O**_**2**_	**M–OOH (Å)**	**M–O**_**face**_ (Å)	**M–O**_**bridge**_ (Å)	**M–O** (Å)	**M–OH** (Å)
	(Δ*G*, eV)	**(Δ*G*, eV)**	**(Δ*G*, eV)**	(Δ*G*, eV)	(Δ*G*, eV)
**Fe**_**5**_ **@Gr**	1.81	1.89,1.90,1.94			1.80
	(3.17 (3.48))	(−0.45 (−0.23))			(−0.22 (−0.23))
**Fe**_**5**_ **@NGr**	1.82		1.78,1.80		1.80
	(2.97 (3.27))		(−0.77 (−0.44))		(−0.27 (−0.30))
**Co**_**5**_ **@Gr**	1.79	1.82,1.84, 2.00			1.77
	(2.86 (3.21))	(−0.4 7 (−0.25))			(−0.45 (−0.46))
**Pt**_**5**_ **@Gr**	1.92			1.78	1.93
	(3.72 (4.13))			(1.05 (1.18))	(0.09 (0.23))

a In parentheses are reported the Δ*G* values in (gas phase­(water implicit solvent)) associated with the formation of *OOH, *O, and *OH intermediates in eV.

**Table 4:** Bond Distance, in Å, between the Active Sites of the Pentamer Metal Clusters and the Adsorbates in the “Unconventional” Intermediates *O*OH and *OH*OH^a^

**1 O**_**2**_	**M–O**_**bridge**_ (Å) M–OH_**bridge**_ (Å) **(Δ*G*, eV)**	**M–OH**_**bridge**_ (Å) M–OH_**bridge**_ (Å) **(Δ*G*, eV)**	**M–OH** (Å) (Δ*G*, eV)
**Fe**_**5**_ **@Gr**	1.78, 1.79	1.93, 1.98	
	1.95, 1.94 (−1.43 (−0.98))	1.96, 1.96 (−1.21 (−0.62))	
**Fe**_**5**_ **@NGr**	1.78, 1.80	1.93, 1.96	
	1.93, 1.97 (−1.62 (−0.34))	1.93, 1.96 (−1.51 (−1.07))	
**Co**_**5**_ **@Gr**	1.75, 1.76	1.87, 1.95	
	1.92, 1.94 (−1.10 (−0.61))	1.86, 1.98 (−1.21 (−0.66))	
**Pt**_**5**_ **@Gr**	1.92, 1.95		1.94, 1.94 (−0.11(0.13))
	2.06, 2.15 (0.65 (1.04))		

a In parentheses are reported the Δ*G* values in (gas phase (water implicit solvent)) associated with the formation of *O*OH and *OH*OH intermediates in eV.

Regardless of the catalyst, the “classical” oxygen reduction passes through the formation of the metastable intermediate *OOH (intermediates A in Figure 2), generated by the interaction of O_2_ with a proton H^+^ and an electron e^–^ (see eq 6 and Figure 2). *OOH reacts with a second H^+^/e^–^ pair, releasing a water molecule and forming intermediate *O (see eq 7 and intermediates B in Figure 2). In the third step, the interaction of *O with a third proton-coupled electron generates the intermediate *OH (see eq 8 and intermediates C in Figure 2), which reacts with a fourth pair of H^+^/e^–^ releasing a second water molecule and restoring the starting catalyst (see eq 9 and Figure 2).

The oxygen reduction reaction can proceed through an alternative pathway to the “classical” one, forming “unconventional” intermediates, i.e., *O*OH and *OH*OH (intermediates A′ and B′ in Figure 2), which energetically compete with the “standard” intermediates *OOH and *O (intermediates A and B in Figure 2). In the “unconventional” mechanism, the interaction of O_2_ with a proton H^+^ and an electron e^–^ generates the intermediate *O*OH (see eq 10 and intermediates A′ in Figure 2), which reacts with a second H^+^/e^–^ pair releasing water, exactly as the classical ORR, but generating an intermediate different from *O, namely, the intermediate *OH*OH (see eq 11 and intermediates B′ in Figure 2). *OH*OH interacts with a third proton-coupled electron forming the “standard” intermediate *OH (see eq 12 and intermediates C in Figure 2). From *OH, the last reduction reaction step proceeds through the “classical” ORR mechanism, previously described (see eq 9 and Figure 2).

The most stable conformations of the “standard” intermediates *OOH involve the coordination of the terminal oxygen of the OOH adsorbates to one of the five metal centers of the *M*_5_ clusters forming M-OOH bonds (intermediates A in Figure 2), whose distances are reported in Table 3.

The intermediates *O are generated by the interaction of one oxygen (O) atom with *M*_5_@Gr­(NGr) catalysts (intermediates B in Figure 2). This interaction can occur through three different coordination modes: Oxygen atom can (i) lie at the center of one face of the trigonal bipyramid (*O*_face_) coordinating three of the five metal centers of the clusters, (ii) bridge between two *M* atoms located on the edge of the trigonal bipyramid (*O*_bridge_) and (iii) form M–O single bonds.

The interaction of the O adsorbate with Fe_5_@Gr and Co_5_@Gr involves the formation of Fe­(Co)-O_face_ bonds (intermediates B in Figure 2a,c and Table 3). In Fe_5_@NGr, only Fe–O_bridge_ bonds are present (intermediate B in Figure 2b and Table 3), whereas in Pt_5_@Gr, the O adsorption generates a single Pt–O bond (intermediate B in Figure 2d and Table 3). Generally, O_face_ bonds are longer than the O_bridge_ ones and both become more labile when the same metal is shared by multiple oxygen atoms (see Table 3). The last intermediate of the “classical” ORR, from which the oxygen reduction product and the starting catalysts are released, is the formation of *OH (intermediates C in Figure 2), in which the OH adsorbate interacts with all considered catalysts through a monocoordination, generating M–OH single bonds whose distances are reported in Table 3.

Moving on to the “unconventional” ORR mechanism, the most stable geometries of the first reaction intermediate *O*OH involve the spontaneous cleavage of the adsorbate O–OH bond, resulting in the formation of one M–O_bridge_ and one M–OH_bridge_ bond (intermediates A′ in Figure 2). This is followed by the formation of the “unconventional” *OH*OH intermediates. In the case of Fe_5_@Gr, Fe_5_@NGr, and Co_5_@Gr catalysts, in the intermediate *OH*OH are present two M–OH_bridge_ bonds (intermediates B′ in Figure 2a–c), whereas with Pt_5_@Gr two single M–OH bonds are formed (intermediates B′ in Figure 2d). For each catalyst, the M–O_bridge_, M–OH_bridge_, and M–OH bond distances related to the *O*OH and *OH*OH intermediates are reported in Table 4.

The Gibbs energy variations (Δ*G*s) related to the formation of the “standard” *OOH intermediates, computed in gas phase and in implicit water solvent, indicate a thermodynamically unstable interaction between the OOH adsorbates and the pentamer metal clusters, whereas the Δ*G*s associated with the *O and *OH intermediates are generally exergonic, indicating a thermodynamically stable interaction between the *M*_5_@Gr­(NGr) catalysts and the O­(OH) adsorbates (see Δ*G* values in Figure 2 and Table 3). Therefore, the first step of the “classical” ORR, i.e., the formation of *OOH intermediates, is the potential determining step (PDS) of the whole O_2_ reduction process.

In Table 4, the Gibbs free energy variations (Δ*G*) associated with the formation of the “unconventional” intermediates *O*OH and *OH*OH are reported. A comparison of the Δ*G* values in Tables 3 and 4 reveals that, both in gas phase and in water, the formation of these “unconventional” intermediates on the *M*_5_@Gr­(NGr) catalysts is thermodynamically more favorable than the formation of the “standard” intermediates *OOH and *O, with energy differences of approximately 4.0–4.5 and 0.7–1.0 eV, respectively.

The data of Tables 3 and 4 highlight that generally the Δ*G* values related to the formation of the intermediates involved in the “classical” and “unconventional” O_2_ reduction process are thermodynamically more stable (Δ*G* < 0) the stronger the interaction between the adsorbates and the pentamer metal clusters. The implicit solvent has a minimal effect on the formation energy of the intermediates involved in the “classical” and “unconventional” ORR mechanisms. Although the Δ*G* values in the solvent are less exergonic (in absolute value) compared with those computed in the gas phase (as shown by the comparison of Δ*G* values in Tables 3 and 4), the overall thermodynamic trends remain unchanged.

Regardless of the mechanism type, stable Δ*G* values are observed for Fe_5_@Gr, Fe_5_@NGr, and Co_5_@Gr catalysts, which exhibit a greater positive total charge due to cluster-surface charge transfer and enhanced orbital availability. However, Fe_5_ cluster coordinates Gr­(NGr) through two­(one) of its five Fe atoms, providing more active sites for interaction with various adsorbates compared to Co_5_, leading to additional structural and electronic stabilization in the formation of both “standard” and “unconventional” intermediates. In contrast, the Pt_5_@Gr catalyst produces ORR intermediates with endergonic Δ*G* values due to electronic factors, such as a high total charge and low orbital availability, destabilizing the interaction between Pt_5_ and the adsorbates.

### Defining the ORR Performance with *M*_5_@Gr­(NGr) Catalysts through the Theoretical Overpotential (η^ORR^) Calculation

To evaluate the ORR performance of the four *M*_5_@Gr­(NGr) catalysts, we computed the theoretical overpotential (η^ORR^) by using eq 13:

13 $$\eta^{\mathrm{ORR}}=U_{0}-U_{L}$$

where *U*_0_ is the equilibrium potential (1.23 V), while *U*_L_, also defined Δ*G*_max_, is the thermodynamic limiting potential of the whole process, that is the highest potential at which all the reaction steps are downhill in free energy.^35,39–42^ The theoretical overpotential can be used as a measure of the catalyst activity: the lower η^ORR^, the more active the catalysts and *vice versa*.

As already described in the previous paragraphs, the ORR proceeds in four steps. Therefore, Δ*G*_max_ corresponds to the highest Δ*G* value associated with each elementary reaction step^40^ and is defined for the “classical” ORR in the eqs 14, 16, and 18, as Δ*G*_1_ (step 1), Δ*G*_2_ (step 2), and Δ*G*_3_ (step 3), and for the “unconventional” ORR in eqs 15, 17, and 19, as Δ*G*_1_′ (step 1), Δ*G*_2_′ (step 2), and Δ*G*_3_′ (step 3). Δ*G*_4_, related to the last reaction step (step 4), is common to both mechanisms, and it is defined in eq 20:

14 ΔG1=ΔGO*OH−4.92eV

15 ΔG1′=ΔGO*O*H−4.92eV

16 ΔG2=ΔGO*−ΔGO*OH

17 ΔG2′=ΔGO*HO*H−ΔGO*O*H

18 ΔG3=ΔGO*H−ΔGO*

19 ΔG3′=ΔGO*H−ΔGO*HO*H

20 ΔG4=−ΔGO*H

In Δ*G*_1_, 4.92 eV is the free energy of O_2_ which corresponds to the experimental value of the Gibbs energy variation associated with the reaction *O*_2_(*g*) + 4H^+^ + 4e^–^ ⇋ 2H_2_O (l).^40,43–47^

Δ*G*_1–4_ are computed for the four *M*_5_@Gr­(NGr) catalysts considering the “standard” and the “unconventional” ORR intermediates. The results are reported in Tables 5 and 6, respectively.

**Table 5:** Δ*G*_1–4_ for the *M*_5_@Gr­(NGr) Catalysts in “Classical” ORR^a^

**1 O**_**2**_	**Fe**_**5**_ **@Gr**	**Fe**_**5**_ **@NGr**	**Co**_**5**_ **@Gr**	**Pt**_**5**_ **@Gr**
Δ*G*_1_	–1.75 (−1.44)	–1.95 (−1.65)	–2.06 (−1.71)	–1.20 (−0.79)
Δ*G*_2_	–3.62 (−3.71)	–3.74 (−3.71)	–3.33 (−3.46)	–2.67 (−2.95)
Δ*G*_3_	**0.23** (0.00)	**0.50** (0.14)	0.02 (−0.21)	–0.96 (−0.95)
Δ*G*_4_	0.22 **(0.23)**	0.27 **(0.30)**	**0.45 (0.46)**	**–0.09 (−0.23)**

a Δ*G* values in the gas phase (water implicit solvent) are in eV and Δ*G*_max_ (*U*_L_) in bold.

**Table 6:** Δ*G*_1–4_ for the *M*_5_@Gr­(NGr) Catalysts in “Unconventional” ORR^a^

**1 O**_**2**_	**Fe**_**5**_ **@Gr**	**Fe**_**5**_ **@NGr**	**Co**_**5**_ **@Gr**	**Pt**_**5**_ **@Gr**
Δ*G*_1_′	–6.35 (−5.90)	–6.54 (−6.26)	–6.02 (−5.53)	–4.27 (−3.88)
Δ*G*_2_′	0.22 (0.36)	0.11 (0.27)	–0.11 (−0.05)	–0.76 (−0.91)
Δ*G*_3_′	**0.99 (0.39)**	**1.24 (0.77)**	**0.76** (0.20)	**0.20 (0.10)**
Δ*G*_4_	0.22 (0.23)	0.27 (0.30)	0.45 **(0.46)**	–0.09 (−0.23)

a Δ*G* values in gas phase (water implicit solvent) are in eV and Δ*G*_max_ (*U*_L_) in bold.

Considering the “classical” oxygen reduction process, the Δ*G*_max_ (*U*_L_) for Fe_5_@Gr and Fe_5_@NGr catalysts is Δ*G*_3_ in gas phase and Δ*G*_4_ in water implicit solvent (see Table 5). Therefore, in the gas phase, the theoretical overpotential η^ORR^ of the two catalysts depends on the binding energy of *OH and *O intermediates, while in solvent, it depends only on the binding energy of the *OH intermediate. Consequently, eq 18 (Δ*G*_3_ = ΔG_*OH_–ΔG_*O_) and eq 20 (Δ*G*_4_ = −ΔG_*OH_) will be the descriptors to predict the ORR activity of these catalysts in the “classical” ORR. For Co_5_@Gr and Pt_5_@Gr catalysts, both in gas phase and in solvent, Δ*G*_max_ is Δ*G*_4_ (see Table 5), indicating a dependence of η^ORR^ on the *OH binding energy (−ΔG_*OH_) (see eq 20).

Unlike the standard mechanism, where Δ*G*_max_ is typically Δ*G*_4_,^35,39^ in the “classical” ORR catalyzed by Fe_5_@Gr and Fe_5_@NGr, an energy stabilization of the *O intermediate occurs compared to the *OH intermediate due to the electronic and structural properties of the clusters (compare the gas phase Δ*G* values of intermediates B and C in Figure 2a,b and in Table 3). Specifically, the formation of the O@Fe_5_@Gr­(NGr) is thermodynamically stabilized by an additional charge transfer from the Fe_5_ clusters to the Gr­(NGr) monolayer of about 0.9(0.8) |e^–^|. Furthermore, the O@Fe_5_@NGr intermediate is more stable than O@Fe_5_@Gr by approximately 0.3 eV, as the Fe–O_bridge_ bonds in O@Fe_5_@NGr are less strained compared to the Fe–O_face_ bonds in O@Fe_5_@Gr (see intermediates B in Figure 2a,b, and Table 3). Therefore, in both cases, the greater stabilization of the *O intermediate compared to that of the *OH intermediate leads to Δ*G*_3_ being defined as the Δ*G*_max_ of the whole process.

When considering the Δ*G* values obtained by using the implicit solvent method, the Δ*G*_max_ of the two Fe catalysts corresponds to Δ*G*_4_ (Table 5). In this case, the implicit water environment exerts a destabilizing effect on the energy of the *O intermediates by about 0.2–0.3 eV, reducing the energy gap between the *O and *OH intermediates. As a result, the thermodynamic limiting potential of the reduction process shifts from Δ*G*_3_ to Δ*G*_4_ (see intermediate B and C in Figure 2a,b, and Δ*G* values in solvent in Tables 3 and 5).

In the case of the “classical” ORR catalyzed by Co_5_@Gr, the energy stability of the O@Co_5_@Gr intermediate is comparable to that of O@Fe_5_@Gr, but not to that of O@Fe_5_@NGr (compare Δ*G* values of the intermediates B in Figure 2a–c and Table 3), due to the strained Fe–O_face_ bonds present in both O@Co_5_@Gr and O@Fe_5_@Gr. In contrast, the thermodynamics related to the formation of the *OH intermediate is more favorable than the corresponding intermediates obtained with the Fe_5_@Gr and Fe_5_@NGr catalysts by about 0.2 eV (compare Δ*G* values of intermediates C in Figure 2a–c and Table 3). Therefore, with Co_5_@Gr, the dependence of the theoretical overpotential on Δ*G*_4_ (Table 5) is related to the stabilization of the *OH intermediate, which arises solely from electronic effects, namely, an additional charge transfer from the metal cluster to the graphene monolayer (about 0.9 |e^–^|).

Also in this case, the implicit solvent causes energy destabilization of the *O intermediate. Despite this, there are no changes in the definition of Δ*G*_max_ as, even in solvent, the OH@Co_5_@Gr intermediate is always more stable than the O@Co_5_@Gr one (compare Δ*G*s of intermediates B and C in Figure 2c and Table 3). Similarly to Co_5_@Gr, also with Pt_5_@Gr, the Δ*G*_max_ in the gas phase and in solvent corresponds to Δ*G*_4_ (Table 5). In this case, the stabilization of the *O intermediates is negligible due to both electronic and structural factors. In fact, the interaction of O adsorbates with the Pt_5_ cluster leads to a charge transfer from the cluster to the monolayer of only 0.5 |e^–^|, and the monocoordination of the O adsorbates to the metal sites does not result in significant structural rearrangements capable of stabilizing the *O intermediates over the *OH ones (see intermediates B in Figure 2d and Δ*G* values in Table 3).

Accordingly, the standard Δ*G*_4_ associated with the “classical” ORR mechanism is observed for Fe_5_@Gr­(NGr) only under solvent conditions, while for Co_5_@Gr and Pt_5_@Gr, it is found both in the gas phase and in solvent.^35,39^

In the “unconventional” ORR mechanism, both in gas phase and in solvent, the Δ*G*_max_ of the four investigated catalysts corresponds to Δ*G*_3_′ due to the large energy gap between the intermediates *OH*OH and *OH (see intermediates B′ and C in Figure 2, Tables 3, 4 and 6). Therefore, in these cases, the theoretical overpotential η^ORR^ depends on the binding energy of the intermediates *OH*OH and *OH (eq 19, Δ*G*_3_′ = ΔG_*OH_–ΔG_*OH*OH_). The only exception is catalyst Co_5_@Gr, whose Δ*G*_max_ in solvent depends on Δ*G*_4_ (eq 20, Δ*G*_4_ = −ΔG_*OH_) (Table 6). This is due to the destabilization effects generated by the implicit solvent which cause a change in the thermodynamic limiting potential of the whole process from Δ*G*_3_ to Δ*G*_4_ (see intermediates A′, B′, and C in Figure 2d, Tables 3, 4, and 6).

In the “unconventional” O_2_ reduction process catalyzed by Fe_5_@Gr, Fe_5_@NGr, and Co_5_@Gr, the thermodynamics associated with the formation of the *O*OH and *OH*OH intermediates is much more favored than that obtained by Pt_5_@Gr, with Fe_5_@NGr returning the most stable formation energies (see intermediates A′ and B′ in Figure 2a–d and Δ*G* values in Table 4).

Generally, the formation of one M–O_bridge_ bond and one M–OH_bridge_ bond, present in the *O*OH intermediates, should be more favored than that of two M–OH_bridge_ bonds, present instead in the *OH*OH intermediates, due to the greater stability of the M-O-M bonds compared to the M-O­(H)-M ones. This hypothesis is in line with the Fe_5_@Gr and Fe_5_@NGr catalysts results, which show Δ*G* of formation values for the *O*OH intermediates more stable than those for the formation of *OH*OH intermediates by about 0.2 and 0.1 eV, respectively (see Δ*G* values and intermediates A′ and B′ in Figure 2a,b and Table 4). Unlike Fe_5_@Gr­(NGr) catalysts and despite the presence of one M–O_bridge_ and one M–OH_bridge_ bond, with Co_5_@Gr, the *O*OH intermediate is less stable than the *OH*OH one by about 0.1 eV (see Δ*G* values in Table 4 and intermediates A′ and B′ in Figure 2c). The higher and/or lower stability of the *O*OH intermediates can also be justified by the different adsorption modes of the Fe_5_ and Co_5_ cluster on the graphene supports: Fe_5_ interacts with Gr­(NGr) through two­(one) of its five metal sites, leaving three­(four) metal centers available for the formation of one M–O_bridge_ bond and one M–OH_bridge_ bond, while Co_5_ engages in the coordination with graphene three of its five metal centers, leaving only two metal sites available for the formation of these bridge bonds. Therefore, in the absence of a higher number of available active sites, which can be shared to form bridge bonds, Co_5_@Gr and Fe_5_@Gr, with two and three available active sites, generate *O*OH intermediates resulting structurally more strained than those obtained with Fe_5_@NGr, which instead has three available active sites.

When ORR occurs on Pt_5_@Gr catalyst, the formation of the “unconventional” *O*OH and *OH*OH intermediates is more favored than that of the “standard” *OOH and *O intermediates thanks to a stabilizing structural rearrangement of the Pt_5_ cluster (compare geometries and energies of the intermediates A, A′ and B, B′ in Figure 2d, Tables 3, and 4).

In this case, the *OH*OH intermediate is energetically more stable than the *O*OH one due to the formation, in *OH*OH, of M–OH single bonds, resulting in less strain than the two bridge bonds present in *O*OH (see intermediates A′ and B′ in Figure 2d and Table 4). However, despite being more stable than the “standard” intermediates *OOH and *O, the Δ*G* values associated with the formation of the “unconventional” intermediates *O*OH and *OH*OH are endergonic or slightly exergonic, precisely because of the high total charge of the cluster and low orbital availability, which destabilize the interaction between Pt_5_ and the adsorbates (see Δ*G* in Table 4).

Generally, in the “unconventional” reduction mechanism the intermediates *O*OH and *OH*OH are energetically favored with respect to the final intermediates *OH (see intermediates A′, B′, and C in Figure 2a–c, Tables 3, and 4), except for Pt_5_@Gr which has *O*OH at about 0.6 eV above the *OH intermediate (see intermediates A′, B′, and C in Figure 2d, Tables 3, and 4).

The theoretical overpotentials (η^ORR^) for the different catalysts, considering the “classical” and “unconventional” reduction mechanism and computed by using eq 13, are reported in Table 7.

**Table 7:** η^ORR^ for the *M*_5_@Gr­(NGr) Catalysts in “Standard” and “Unconventional” ORR^a^

**“classical” ORR**	**Fe**_**5**_ **@Gr**	**Fe**_**5**_ **@NGr**	**Co**_**5**_ **@Gr**	**Pt**_**5**_ **@Gr**
η^ORR^	1.0(1.0)	0.7(0.9)	0.8(0.8)	1.3(1.5)

**“unconventional” ORR**	**Fe**_**5**_ **@Gr**	**Fe**_**5**_ **@NGr**	**Co**_**5**_ **@Gr**	**Pt**_**5**_ **@Gr**
η^ORR^	0.2(0.8)	0.0(0.5)	0.5(0.8)	1.0(1.1)

a Δ*G* values in gas phase (water implicit solvent) are in V.

Unlike the experimental overpotential, which is governed by kinetic factors, the theoretical overpotential (η^ORR^) is a parameter based on the thermodynamics of the intermediates involved in a chemical process, in this case, the O_2_ reduction. The theoretical overpotential is widely used to evaluate and compare the catalytic performance of heterogeneous catalysts as it serves as a measure of catalytic activity.

Starting from the assumption that lower the η^ORR^, the more active the catalyst, and *vice versa*,^35,48^ the results reported in Table 7 allow us to describe the catalytic performances of the four catalysts in the “classical” and “unconventional” ORR.

In the “classical” reduction process, Fe_5_@Gr, Fe_5_@NGr, and Co_5_@Gr show good catalytic activity, with theoretical overpotentials values between 0.7 and 1.0 V, while Pt_5_@Gr exhibits the worst behavior. In fact, with overpotential values of 1.3 V in the gas phase and 1.5 V in the solvent, it turns out to be the least active catalyst among those investigated (see η^ORR^ values in the gas phase and in the solvent reported in the second row of Table 7).

The catalytic performances of Fe_5_@Gr and Fe_5_@NGr significantly improve in the “unconventional” ORR, where the two catalysts reduce their theoretical overpotential by 0.8 and 0.7 V in gas phase and by 0.2 and 0.4 V in solvent, respectively. The η^ORR^ reduction suggests a high stability and a better activity of the two systems (see η^ORR^ values in gas phase and in solvent reported in the fourth row of Table 7). In the “unconventional” mechanism, the catalytic efficiency of Co_5_@Gr relatively increases. In fact, moving from the “classical” to the “unconventional” ORR, the theoretical overpotential of the catalyst is reduced by 0.3 V in gas phase, remaining unchanged in solvent. In “unconventional” conditions, the catalytic performance of Pt_5_@Gr also improves, with its theoretical overpotential reduced by 0.3 V in gas phase and 0.4 V in solvent (see η^ORR^ values in gas phase and in solvent reported in the fourth row of Table 7).

Therefore, based on the results reported in Table 7, we can conclude that, both in the “classical” and “unconventional” ORR, all of the investigated catalysts have good stability and relatively high activities. Furthermore, while in the “classical” mechanism the four catalysts compete in terms of activity, in the “unconventional” condition, the most promising catalyst is Fe_5_NGr, followed by Fe_5_@Gr, Co_5_@Gr, and Pt_5_@Gr.

## Conclusions

In this work, DFT calculations were carried out to provide information about the catalytic activity of graphene-supported pentamer metal clusters (Fe_5_, Co_5_, and Pt_5_) in the ORR processes, under standard electrochemical conditions (pH = 0 and *U* = 0) and considering both the gas phase and implicit water solvent.

Energy and structural analyses confirm the thermodynamic stability of the *M*_5_@Gr­(NGr) catalysts, with negative binding and cohesion energy values in both environments, suggesting that Fe_5_@NGr is the most stable catalyst. In this case, doping of the graphene monolayer with N atoms creates a vacancy site well suited for interaction with the Fe_5_ cluster, allowing for coordination with its available low-energy orbitals. The direct coordination of an iron atom with the four N-doped sites results in high electronic delocalization and significant charge transfer to the support, reaching up to 1.8 |e^–^|.

Along with the “classical” ORR mechanism, involving the intermediates *OOH, *O, and *OH, an “unconventional” path was also studied, leading to the formation of *O*OH and *OH*OH intermediates, which are thermodynamically more stable both in gas phase and solvent. This suggests a preference for the “unconventional” mechanism by the *M*_5_@Gr­(NGr) catalysts.

The catalytic performances of each catalyst were evaluated through quantitative assessment of the theoretical overpotential (η^ORR^). In the “classical” reduction process, Fe_5_@Gr, Fe_5_@NGr, and Co_5_@Gr exhibit good catalytic activity, with relatively low theoretical overpotential values. Pt_5_@Gr, with slightly higher overpotential values, shows the least catalytic activity among the studied catalysts. The catalytic efficiencies of the *M*_5_@Gr­(NGr) systems improve in the “unconventional” mechanism, where further reductions in the theoretical overpotential are observed. In this case, Fe_5_@NGr exhibits the best catalytic performance, with η^ORR^ approaching zero.

In conclusion, while in the “classical” mechanism the four catalysts demonstrate comparable activity, in the “unconventional” path, Fe_5_@NGr emerges as the most promising catalyst, followed by Fe_5_@Gr, Co_5_@Gr, and Pt_5_@Gr. Despite the potential limitations posed by the high stability of the “unconventional” intermediates, our work provides valuable insights for the rational design of low-cost graphene-supported metal cluster catalysts. These results contribute to a better understanding of how to modify the electronic properties of the clusters and supports to enhance the stability and efficiency of such catalysts in the ORR process.

## Supplementary Material



## Notes

Equations to compute the Gibbs energy variation (Δ*G*, eV) of each “standard” and “unconventional” intermediate of the ORR process, the ZPE corrections for H_2_ and H_2_O gas phase species and for the different species adsorbed on *M*_5_@Gr­(NGr) (*M* = Fe, Pt, Co), and the entropy values of the H_2_ and H_2_O species at room temperature (PDF)
