# Supplementary material for: Advances in ORR Catalysis Promoted by Graphene-Supported Low-Cost Metal Clusters: A DFT Study
Source: ACS Appl Mater Interfaces. 2025 Jun 16;17(27):39708–18. doi: 10.1021/acsami.5c08441 (PMC12257453; doi:10.1021/acsami.5c08441)
Supplement: Supplementary file 1 [file am5c08441_si_001.pdf]

Supporting Information

Advance on ORR Catalysis Promoted by Low-Cost Metal Clusters Graphene-Supported: A DFT Study

Ida Ritacco,[a] Giuseppe Santoriello,[a] Matteo Farnesi Camellone, [b]Lucia Caporaso*[a]*

[a] Dipartimento di Chimica e Biologia, Università degli Studi di Salerno, via Giovanni Paolo II 132, 84084 Fisciano, Salerno, Italy. E-mail: iritacco@unisa.it; lcaporaso@unisa.it.

[b] CNR-IOM, Consiglio Nazionale delle Ricerche - Istituto Officina dei Materiali, c/o SISSA, 34136 Trieste, Italy.

KEYWORDS: Oxygen reduction reaction (ORR), heterogeneous catalysis, clusters, graphene, DFT, N-doped graphene, theoretical overpotential (η)

Section 1. Equations to compute the Gibbs energy variations (ΔGs, eV) associated with the 'standard' and 'unconventional' ORR intermediates.

In this section we report the equations (1-5) to compute the Gibbs energy related to the adsorption of the 'standard' and 'unconventional' ORR intermediates on M_5@Gr(NGr) (M=Fe, Pt, Co) catalysts at standard conditions (T=298.15 K, U=0 and pH=0). M_5@Gr(NGr) is labelled as *.

1. Equation for the OOH intermediate on M_5@Gr(NGr) (*)

$$* + 2H_2O \rightarrow *OOH + \frac{3}{2} H_2$$

ΔE(OOH) = $E_{*OOH} + \frac{3}{2} E_{H2} – E_{*} - 2E_{H2O}$

ΔZPE(OOH) = $ZPE_{*OOH} + \frac{3}{2} ZPE_{H2} - 2ZPE_{H2O}$

TΔS(OOH) = $\frac{3}{2} TS_{H2} - 2TS_{H2O}$

ΔE_{SOLV}(OOH) = $E_{SOLV\_*OOH} + \frac{3}{2} E_{SOLV\_H2} – E_{SOLV\_*} - 2E_{SOLV\_H2O}$

ΔG (OOH) = ΔE(OOH) + ΔZPE(OOH) - TΔS(OOH) + ΔE_{SOLV}(OOH)

2. Equation for the O intermediate on M_5@Gr(NGr) (*)

$$* + H_2O \rightarrow *O + H_2$$

ΔE(O) = $E_{*O} + E_{H2} – E_{*} - E_{H2O}$

ΔZPE(O) = $ZPE_{*O} + ZPE_{H2} - ZPE_{H2O}$

TΔS(O) = $TS_{H2} - TS_{H2O}$

ΔE_{SOLV}(O) = $E_{SOLV\_*O} + E_{SOLV\_H2} – E_{SOLV\_*} - E_{SOLV\_H2O}$

ΔG (O) = ΔE(O) + ΔZPE(O) - TΔS(O) + ΔE_{SOLV}(O)

3. Equation for the OH intermediate on M_5@Gr(NGr) (*)

$$* + H_2O \rightarrow *OH + \frac{1}{2} H_2$$

ΔE(OH) = $E_{*OH} + \frac{1}{2} E_{H2} – E_{*} - E_{H2O}$

ΔZPE(OH) = $ZPE_{*OH} + \frac{1}{2} ZPE_{H2} - ZPE_{H2O}$

$T\Delta S(OH)$ = $\frac{1}{2}$ TS_{H2} - TS_{H2O}

$\Delta E_{SOLV}(OH)$ = E_{SOLV_*OH} + $\frac{1}{2}$ E_{SOLV_H2} – E_{SOLV_*} - E_{SOLV_H2O}

ΔG (OH) = $\Delta E(OH)$ + $\Delta ZPE(OH)$ - $T\Delta S(OH)$ + $\Delta E_{SOLV}(OH)$

4. Equation for the O-OH intermediate on M_5@Gr(NGr) (*)

$$* + 2H_2O \rightarrow *O*OH + \frac{3}{2} H_2$$

$\Delta E(O\text{-}OH)$ = E_{*O*OH} + $\frac{3}{2}$ E_{H2} – E_* - $2E_{H2O}$

$\Delta ZPE(O\text{-}OH)$ = ZPE_{*O*OH} + $\frac{3}{2}$ ZPE_{H2} - $2ZPE_{H2O}$

$T\Delta S(O\text{-}OH)$ = $\frac{3}{2}$ TS_{H2} - $2TS_{H2O}$

$\Delta E_{SOLV}(O\text{-}OH)$ = E_{SOLV_*O*OH} + $\frac{3}{2}$ E_{SOLV_H2} – E_{SOLV_*} - $2E_{SOLV\_H2O}$

ΔG (O-OH) = $\Delta E(O\text{-}OH)$ + $\Delta ZPE(O\text{-}OH)$- $T\Delta S(O\text{-}OH)$ + $\Delta E_{SOLV}(O\text{-}OH)$

5. Equation for the OHOH intermediate on M_5@Gr(NGr) (*)

$$* + 2H_2O \rightarrow *OH*OH + H_2$$

$\Delta E(OHOH)$ = E_{*OH*OH} + E_{H2} – E_* - $2E_{H2O}$

$\Delta ZPE(OHOH)$ = ZPE_{*OH*OH} + ZPE_{H2} - $2ZPE_{H2O}$

$T\Delta S(OHOH)$ = TS_{H2} - $2TS_{H2O}$

$\Delta E_{SOLV}(OHOH)$ = E_{SOLV_*OH*OH} + E_{SOLV_H2} – E_{SOLV_*} - $2E_{SOLV\_H2O}$

ΔG (OHOH) = $\Delta E(OHOH)$ + $\Delta ZPE(OHOH)$ - $T\Delta S(OHOH)$ + $\Delta E_{SOLV}(OHOH)$

Table S1. ZPE corrections for a) H_2 and H_2O gas phase species and for b) the 'standard' and 'unconventional' ORR intermediates. Values are reported in eV.

a)

	H_2	H_2O
ZPE	0.27	0.55

b)

O_2	ZPE_{OOH*}	ZPE_{O*}	ZPE_{OH*}	ZPE_{*O*OH}	ZPE_{*OH*OH}
Fe_5@Gr	0.87	0.37	0.64	0.90	1.18
Fe_5@NGr	0.76	0.31	0.67	0.96	1.30
Co_5@Gr	0.69	0.18	0.52	0.82	1.11
Pt_5@Gr	0.98	0.06	0.32	0.38	0.47

Table S2. Entropy values of the H_2 and H_2O species at room temperature. Values are reported in eV.

	H_2	H_2O
TS	0.41	0.58
